# Surprise, surprise: KK is innocent

**DOI:** 10.1002/tht3.473

**Published:** 2021-02-16

**Authors:** Julien Murzi, Leonie Eichhorn, Philipp Mayr

**Affiliations:** ^1^ Philosophy Department KGW University of Salzburg Salzburg Austria

## Abstract

The Surprise Exam Paradox is well‐known: a teacher announces that there will be a surprise exam the following week; the students argue by an intuitively sound reasoning that this is impossible; and yet they *can* be surprised by the teacher. We suggest that a solution can be found scattered in the literature, in part anticipated by Wright and Sudbury, informally developed by Sorensen, and more recently discussed, and dismissed, by Williamson. In a nutshell, the solution consists in realising that the teacher's announcement is a *blindspot* that can only be known if the week is at least 2 days long. Along the way, we criticise Williamson's own treatment of the paradox. In Williamson's view, the Surprise is similar to the Paradox of the Glimpse and, because of their similarities, both these paradoxes ought to receive a uniform treatment—one that involves locating an illicit application of the KK Principle. We argue that there's no deep analogy between the Surprise and the Glimpse and that, even if there were, the Surprise reasoning reaches a paradoxical conclusion *before* the KK Principle is used. Rather, in both the Surprise and the Glimpse, the blame should be put on other epistemic principles—respectively, a knowledge retention and a margin for error principle.

The Surprise Exam Paradox seemingly shows that a teacher cannot give a surprise test to her students. For, after all, if it was given on the last day of the week, they would know on the previous day that it would be given then, and the exam would no longer be a surprise. Similarly, they rule out every other day of the week: if the exam was given on Thursday, they would know on Wednesday that it would be given then, and the exam would no longer be a surprise, and so on. Yet, it would also seem, the teacher *can* surprise the students. Where does the students' reasoning go wrong?

We suggest that a solution can be found scattered in the literature, in part anticipated by Wright and Sudbury (1977), informally developed by Sorensen (1988), and more recently discussed, and dismissed, by Williamson (2000, Ch. 6). In a nutshell, the solution consists in realising that the teacher's announcement is a *blindspot* that can only be known if the week is at least 2 days long, whence a surprise exam can be given on any day of the week and the students are mistaken in ruling out the last day of the week as a possible exam day. It follows from our diagnosis that Tim Williamson's contention that the surprise reasoning relies on an illicit application of the KK Principle (KK)—that if *S* knows *P*, then she knows that she knows *P*—is actually off target. Williamson (2000, Ch. 6) claims that the Surprise Exam Paradox is similar to a paradox based on a margin for error principle—the Paradox of the Glimpse—and that since both reasonings involve KK this must be were both reasonings break down. However, as we argue, invalidating the KK step in the Surprise is both of (i) *no use* and (ii) *no need*. *Ad (i)*, we show that a paradoxical conclusion is reached *before* KK is applied in the course of the paradoxical reasoning. *Ad (ii)*, as both Wright and Sudbury (1977) and Sorensen (1988) point out, the students' reasoning provably fails because of the inconsistency between a certain knowledge retention principle and the existence of blindspots for knowledge. Indeed, we suggest, there's no deep analogy between the Glimpse and the Surprise: they are different reasonings involving different assumptions as well as different epistemic principles. What's more, the grounds for invalidating KK in the Glimpse are weak, since, we submit, there's independent reasons for doubting the validity of the relevant margin for error principle.^1^

## 1 THE STUDENTS' REASONING

Following Kripke (2011, p. 30 and ff), we formalise the teacher's announcement as the conjunction of three claims: that an exam will be given between Monday and Friday (of the week after the announcement is made), that the exam will be given on exactly 1 day, and that the exam will be a *surprise*, in the sense that the day prior to the exam the students do not know that the exam will take place on the next day.^2^

More formally:

(K1) *E*_*i*_ for some *i*, 1 ≤ *i* ≤ 5 (equivalently: *E*_1_ ∨ , …, ∨ *E*_5_).
(K2) ¬(*E*_*i*_ ∧ *E*_*j*_) for any i=j,1≤i,j≤5.
(K3) $\neg K_{i-1}E_{i}$ for each *i*, 1 ≤ *i* ≤ 5.

For simplicity, we associate each week day with a natural number, starting from Friday (the week in which the exam is announced) = 0, Monday (the week in which the exam takes place) = 1, and so on. We tacitly take K2 for granted and formalise the teacher's announcement as the conjunction $E_{i}\wedge \neg K_{i-1}E_{i}$.^3^ Still following Kripke, we assume that if the exam has not been given on the first *i* − 1 days, then the students know this on day *i* − 1 and that if the exam is given on day *i*, then the students know on day *i* − 1 that it has not been given on any of the first *i* − 1 days:

$$\left(K4\right)\frac{\neg E_{1}\wedge \neg E_{2}\wedge .\ldots \wedge \neg E_{i-1}}{K_{i-1}\left(\neg E_{1}\wedge \neg E_{2}\wedge \ldots \wedge \neg E_{i-1}\right)}\;\left(K5\right)\frac{E_{i}}{K_{i-1}\left(\neg E_{1}\wedge \neg E_{2}\wedge .\ldots \wedge \neg E_{i-1}\right)}$$

We then make some standard assumptions about knowledge: that knowledge is factive, that is, that we only know truths, and that knowledge is closed under known material implication, that is, that if the students know *P* on day *i* and know on that day that, if *P*, then *Q*, then they also know *Q* on day *i*. To simplify derivations, we also directly assume that knowledge distributes over known conjunctions. More formally:

$$\left(F\right)\frac{K_{i}P}{P}\;\left(\mathrm{EC}\right)\frac{K_{i}P\;K_{i}\left(P\to Q\right)}{K_{i}Q}\;\left(D\right)\frac{K_{i}\left(A\wedge B\right)}{K_{i}A\wedge K_{i}B}$$

Although EC may be thought to be problematic in general, the relevant instances in the surprise reasoning should be beyond reproach: the students' reasoning only involves a small number of sentences and only requires a couple of very innocent instances of EC.

Most of our discussion focuses on a knowledge retention principle, to the effect that if the students know *P* on a given day *i*, they know *P* on any later day *j*, and on the KK principle, that if the students know *P* on a given day, then they know on that day that they know *P* on that day:

$$\left(\mathrm{KR}\right)\;\frac{K_{i}P}{K_{j}P}\;\left(\mathrm{KK}\right)\frac{K_{i}P}{K_{i}K_{i}P}$$

Now let T be any sentence provable in the epistemic logic given by the above principles—call such as logic Logic^+^. We finally assume that the students know such a logic on each of the relevant days:

$$\left(\mathrm{LO}\right)\frac{T}{K_{i}\left(T\right)}$$

Again, while LO may be false in general, only a couple of unproblematic instances are required in the derivation of the paradox. We are now in a position to precisely regiment the students' reasoning.

We assume that the exam is announced on day 0, that is, Friday the week before the week the exam is meant to take place. We then derive, on the assumption that the exam will take place the following Friday, that the students know on Thursday that the exam will take place on Friday. For reasons of space, we abbreviate the claim that no exam takes place between Monday and Thursday as ¬*E*_1 − 4_:


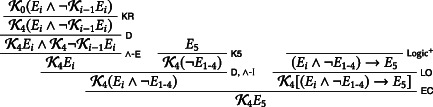


Call the above derivation, with open assumptions $K_{0}\left(E_{i}\wedge \neg K_{i-1}E_{i}\right)$ and *E*_5_, $D_{0}$. In our next step, we use $D_{0}$ to show that the exam will not take place on Friday:


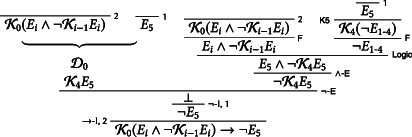


Call this second derivation $D_{1}$. We now use $D_{1}$ and KK to prove that the students know at the outset that the exam will not be on Friday:

$$\frac{\frac{K_{0}\left(E_{i}\wedge \neg K_{i-1}E_{i}\right)}{K_{0}\left[K_{0}\left(E_{i}\wedge \neg K_{i-1}E_{i}\right)\right]}\mathrm{KK}\;\begin{array}{c} D_{1}\\ \frac{K_{0}\left(E_{i}\wedge \neg K_{i-1}E_{i}\right)\to \neg E_{5}}{K_{0}\left[K_{0}\left(E_{i}\wedge \neg K_{i-1}E_{i}\right)\to \neg E_{5}\right]}\mathrm{LO} \end{array}}{K_{0}\neg E_{5}}\mathrm{EC}$$

To conclude that the students know, paradoxically, that no surprise exam can take place between Monday and Friday, we repeat versions of the above argument four more times (assuming *E*_4_, *E*_3_, and so on). Where does the argument go wrong?

Quine (1953) famously suggested that the teacher's announcement is *never known*. Thus, $K_{0}\left(E_{i}\wedge \neg K_{i-1}E_{i}\right)$ cannot feature as a premise in the students' reasoning, and the paradoxical reasoning never gets off the ground. In particular, the base step of the students' reasoning—their elimination of Friday as a possible exam day—is already mistaken. However, Quine's solution comes at a heavy price. Suppose the teacher's announcement is true and the teacher is trustworthy and reliable. Then, why should not the students come to *know* that there will be a surprise exam that week on the basis of the testimony offered by their trustworthy and reliable teacher? If the students cannot know their teacher's announcement, by parity of reasoning, there is very little, if anything, they can know by testimony. But such an extreme form of scepticism about testimony is hardly palatable.

Less implausibly, Kripke (2011) takes KR, the knowledge retention principle, to be the culprit. In Kripke's view, as the examination less days pass, the students *start doubting* the truth of the teacher's announcement and finally lose knowledge of the announcement. However, this also seems problematic. If the teacher is known to be trustworthy and reliable, it is unclear whether the students' confidence can be eroded in just a few days. KR may well be the culprit, but Kripke's explanation *why* KR is to blame fails to convince.

Williamson (2000, Ch. 6) recommends instead blaming the students' reliance on KK. In his view, the surprise reasoning belongs to a family of epistemic paradoxes all of which are most plausibly invalidated by disallowing certain applications of KK. We'll say more about Williamson's take on the paradox in §§3–4 below. For the time being, we simply notice that the instances of KK that are required in the students' reasoning are all fairly uncontroversial, and should be expected to hold if the teacher is known for her trustworthiness and reliability (cf. Kripke, 2011, pp. 34–35).^4^

Quine's and Kripke's solutions come closer to the mark, even if not close enough. Both Quine and Kripke correctly locate the fallacy in the mistaken assumption that the teacher's announcement can be known on each day of the week. However, neither Quine nor Kripke offer an adequate explanation *why* the students cannot always have knowledge of the teacher's announcement. To this explanation we now turn.

## 2 BLINDSPOTS

We largely follow, and for the first time formally regiment, ideas informally presented in Sorensen (1988, Ch. 9). We begin by showing that, if the teacher's announcement is known on Monday and there's no exam by Thursday, then, courtesy of the knowledge retention principle KR, the students know on Thursday that there will be a surprise exam on Friday.

Lemma 1 *Let* *S* *be a theory strong enough to validate* $K_{0}\left(E_{i}\wedge \neg K_{i-1}E_{i}\right)$, K4, EC, and LO. *Then*, *S* *validates KR only if it also validates* $K_{4}\left(E_{5}\wedge \neg K_{4}E_{5}\right)$.

Let $D_{2}$ be the following derivation:

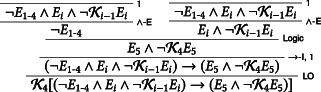

We use $D_{2}$ to prove that the students know $E_{5}\wedge \neg K_{4}E_{5}$ on Thursday:

$$\frac{\begin{array}{c} D_{2}\\ K_{4}\left[\left(\neg E_{1-4}\wedge E_{i}\wedge \neg K_{i-1}E_{i}\right)\to \left(E_{5}\wedge \neg K_{4}E_{5}\right)\right] \end{array}\;\frac{\frac{\frac{\neg E_{1-4}}{K_{4}\left(\neg E_{1-4}\right)}\mathrm{K4}\;\frac{K_{0}\left(E_{i}\wedge \neg K_{i-1}E_{i}\right)}{K_{4}\left(E_{i}\wedge \neg K_{i-1}E_{i}\right)}\mathrm{KR}}{K_{4}\neg E_{1-4}\wedge K_{4}\left(E_{i}\wedge \neg K_{i-1}E_{i}\right)}\wedge -I}{K_{4}\left(\neg E_{1-4}\wedge E_{i}\wedge \neg K_{i-1}E_{i}\right)}D}{K_{4}\left(E_{5}\wedge \neg K_{4}E_{5}\right)}\mathrm{EC}$$

We now show, following a well‐known reasoning due to Alonzo Church and first published in Fitch (1963), that $K_{4}\left(E_{5}\wedge \neg K_{4}E_{5}\right)$ leads to inconsistency given D and F.^5^

Theorem 2 *Let* *S* *be a theory strong enough to validate F, D, and* $K_{4}\left(E_{5}\wedge \neg K_{4}E_{5}\right)$. *Then*, *S* *derives* ⊥.

We assume $K_{4}\left(E_{5}\wedge \neg K_{4}E_{5}\right)$ and make use of the principles D and F:
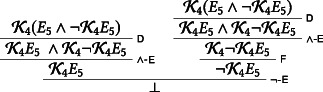

Corollary 3 *S* *validates* $K_{1}\left(E_{i}\wedge \neg K_{i-1}E_{i}\right)$ *, K4, EC, D, F, LO, and KR, only if it derives* ⊥.

Immediate from Lemma 1 and Theorem 2.

So much for the technical results.

Corollary 3 shows that the epistemic principles the students rely on, together with the factivity of knowledge, are inconsistent. Thus, something has to give. But what? None of $K_{0}\left(E_{i}\wedge \neg K_{i-1}E_{i}\right)$, K4, EC, D, F, and LO can be reasonably doubted in the present context. To repeat, to give up $K_{0}\left(E_{i}\wedge \neg K_{i-1}E_{i}\right)$ is to give into an unacceptable, and unjustified, scepticism about testimony; K4, D, F are beyond reproach; and while EC and LO may be false in general, the relevant instances required in order to run the students' reasoning cannot be seriously questioned.

On these assumptions, there is only one possible culprit left: KR. The natural lesson to learn from the paradox, then, is that the students cannot in general retain knowledge of the teacher's announcement throughout the week. In particular, they must lose such a knowledge on Thursday, on pain of inconsistency. As Williamson himself puts it:

[T]o know on the last day that there will be a surprise examination, when there has been none so far, is in effect to know “There will be an examination tomorrow and we do not know that there will be an examination tomorrow”. Such knowledge is impossible, for their knowledge of the first conjunct is inconsistent with the truth of the second […]. Thus if the examination is on the last day, then the pupils will have lost their knowledge of the truth of the teacher's announcement by the last morning. (Williamson, 2000, p. 138)

We return to Williamson's assessment of the present diagnosis in §4 below. For the time being, we notice that Lemma 1 clearly explains what goes wrong in the derivation of the paradox presented in the previous section. In particular, the KR step in the proof of Lemma 1, viz. the step from $K_{0}\left(E_{i}\wedge \neg K_{i-1}E_{i}\right)$ to $K_{4}\left(E_{i}\wedge \neg K_{i-1}E_{i}\right)$, also occurs in the very first two lines of derivation $D_{0}$ in the previous section. Since, as we have just seen, such a step is not in general truth‐preserving, we have no reason to think that it preserves truth in $D_{0}$. That is, the proof of Lemma 1 already reveals what goes wrong in the students' reasoning, without any need to invoke KK.

## 3 KK IS INNOCENT

The students' reasoning seemingly establishes—without making use of KK—that the surprise exam cannot take place on Friday. Yet the exam *can* take place on Friday: the teacher might of course decide to give it then. And it can also be a surprise on Friday. If no exam has been given yet on Thursday, it is an immediate consequence of Theorem 2 that the students can no longer know then the teacher's announcement. But if (i) the students do not know on Thursday that there will be a surprise exam on Friday and (ii) an exam is given on Friday, the students are surprised on Friday, given the definition of surprise.^6^ Thus, if the exam is given on Friday, the students reach a false conclusion—namely, that there will not be an exam on Friday—without making use of KK. Invalidating KK is therefore of *no use*: it does nothing to invalidate the core of the students' invalid reasoning. Williamson would need to argue that the exam cannot be given on Friday. But this would be a bad move: the teacher's announcement says that a surprise exam will be given between Monday *and Friday*, and of course the teacher can give the exam on Friday, if she so wishes.

However, it should already be clear that invalidating KK is also of *no need*. To see this, notice that the students' reasoning proceeds by assuming, for *reductio*, that the exam will be given on Friday. And, as we have already observed in §2, the subproof opened by the assumption that the exam will take place on Friday involves a step of KR, from $K_{0}\left(E_{i}\wedge \neg K_{i-1}E_{i}\right)$ to $K_{4}\left(E_{i}\wedge \neg K_{i-1}E_{i}\right)$. Using such a step, the students derive a contradiction and proceed to negate, and discharge, the assumption that the exam will take place on Friday, thereby eliminating Friday as a possible exam date. However, as we have seen, Lemma 1 shows that, if no exam has been given by Thursday, *that very same step of* KR commits the students to knowing something they cannot know: that an exam will be given on Friday and that they do not know that an exam will be given on Friday. But, as we know from Theorem 2, this is impossible. Thus, KR is false: knowledge of the teacher's announcement cannot be retained on Thursday, if no exam has been given by then. More precisely, the step of KR used at the very beginning of the students' reasoning, viz. lines 1 and 2 in $D_{0}$, is invalid, and it is therefore a mistake to close the subproof opened by the assumption that the exam will take place on Friday by negating and discharging such an assumption: KR should be faulted instead.

It might be objected that the students' reasoning *need not* be reconstructed as involving a commitment, via KR, to $K_{4}\left(E_{5}\wedge \neg K_{4}E_{5}\right)$. To wit, consider the following version of $D_{0}$, call it $D_{0}^{\prime }$:
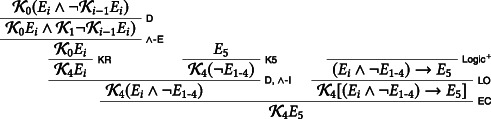


Clearly, $D_{0}^{\prime }$ does not involve the problematic step of KR, viz. the step from $K_{0}\left(E_{i}\wedge \neg K_{i-1}E_{i}\right)$ to $K_{4}\left(E_{i}\wedge \neg K_{i-1}E_{i}\right)$. The students' reasoning can now go on as usual: they derive $\neg K_{4}E_{5}$, reach a contradiction, and thereby negate and discharge their initial assumption *E*_5_. Thus, one might insist, the students are *perfectly justified* to rule out Friday: this does not commit them to knowing blindspots for knowledge.

The objection trades on a subtle epistemic fallacy, however. Theorem 2 establishes that the students can no longer know the teacher's announcement on Thursday, if no exam has been given by then. Notice, though, that the teacher's announcement is a *conjunction*: that there will be an exam *and* that it will be a surprise. Now, it is consistent with the proof of Theorem 2 that the students know either conjunct on Thursday without knowing the other. This is precisely what happens in $D_{0}^{\prime }$: here on Thursday the students know *E*_*i*_, but do not know $\neg K_{4}E_{i}$. However, the envisaged objection offers no positive story as to why the students come to privilege knowledge of *E*_*i*_ at the expenses of knowledge of $K_{4}E_{i}$, when *ex hypothesi* they have acquired knowledge of *both* conjuncts via the teacher's announcement on Friday the week before the exam is meant to take place. As we have seen, such an announcement can no longer be known the following Thursday. But then, when knowledge of the teacher's announcement is lost on Thursday, it is completely arbitrary to insist that the students can still know one conjunct at the expenses of the other, when the students have no reasons for believing one part of the teacher's announcement more strongly than the other (see also Sorensen, 1988, p. 330).^7^

## 4 THE SURPRISE AND THE GLIMPSE

Sorensen (1988, Ch. 9) advocates something like the strategy we have just sketched as a solution to the Surprise and related paradoxes. Williamson also shows some degree of sympathy. He writes:

[T]he reasoning by which [the students] rule out a last‐day examination is unsound, for it assumes that knowledge will be retained in trying to refute a supposition on which it would not be retained. The foregoing diagnosis can be elaborated in a variety of ways. There is clearly something to it. (Williamson, 2000, p. 138)

However, Williamson dismisses the blindspot diagnosis as “incomplete” and ultimately mistaken, on the grounds that it does not extend to what Williamson takes to be a simpler version of the Surprise—what he calls the *Glimpse*. Williamson introduces the Glimpse thus:

A teacher's pupils know that she rings all and only examination dates on the calendar in her office. At the beginning of term, the only knowledge they have of examination dates this term comes from a distant glimpse of the calendar, enough to see that one and only one date is ringed and that it is not very near the end of term, but not enough to narrow it down much more than that. The pupils recognize their situation. They know now that for all numbers *i*, if the examination is *i* + 1 days from the end of term then they do not know now that it will not be *i* days from the end (*o* ≤ *i* ≤ *n*). In particular, they know now that if it is on the penultimate day then they do not know now that it will not be on the last day. But they also know now from their glimpse of the calendar that it will not be on the last day. They deduce that it will not be on the penultimate day. They also know now that if it is on the antepenultimate day then they do not know now that it will not be on the penultimate day. They rule out every day of term as possible date for the examination. (Williamson, 2000, p. 135)

Both the Surprise and the Glimpse are fallacious pieces of reasoning in which all the days in a given interval are mistakenly ruled out as possible exam dates. Williamson takes this analogy to be strong enough to require that the two puzzles be given a similar solution. And, he argues, since the Glimpse involves no blindspots for knowledge, the blindspot approach to the Surprise is not fully general, and hence cannot be correct. As he puts it:

[The blindspots] analysis […] is incomplete. It yields no objection to the reasoning in the Glimpse, which is an equally unsound simplification of the reasoning in the Surprise Examination. What is wrong in the Glimpse is wrong in the Surprise Examination too, yet unmentioned in the diagnosis. (Williamson, 2000, p. 138)

That is, Williamson maintains that what should be mentioned in the diagnoses of both the Surprise and in the Glimpse is the students' reliance on KK. To see this, we first need to present the Glimpse in some more detail.

The Glimpse makes use of the following *margin for error principle* (where $K_{0}$ expresses the students' knowledge after their glimpse of the calendar but prior to the beginning of the term):

$$E_{i+1}\to \neg K_{0}\neg E_{i}$$

This says that if the exam is *i* + 1 days from the end of term, then the students do not know at the outset that it is not *i* days from the end of term. That is, if the exam is *i* + 1 days from the end of term, then for all the students know at the outset, the exam could well be *i* days from the end of term. Equivalently, if the students know at the outset that the exam is not on day *i* from the end of term, then the exam is not on day *i* + 1 from the end of term:

$$K_{0}\neg E_{i}\to \neg E_{i+1}$$

In Williamson's view, the principle holds whenever we have *inexact knowledge*—for instance, knowledge that an exam will be given at some point next term, or that someone's height is roughly two meters. It is motivated by an essentially reliabilist, safety‐based conception of knowledge—one according to which if one knows that *P*, then one's belief that *P* could not have easily been wrong (see for example, Williamson, 2000, Ch. 5). Now let *l* and *p* be the last and the penultimate days of term, respectively. Let us further assume, with Williamson, that the students know at the outset that a margin for error is actually in play, that is, they know at the outset $K_{0}\neg E_{i}\to \neg E_{i+1}$. We can then represent the students' reasoning in the Glimpse as follows:

$$\frac{K_{0}\left(K_{0}\neg E_{l}\to \neg E_{p}\right)\;\frac{K_{0}\neg E_{l}}{K_{0}K_{0}\neg E_{l}}\mathrm{KK}}{K_{0}\neg E_{p}}\mathrm{EC}$$

Let *n* be the number of possible exam dates during the term. We repeat the reasoning *n* − 1 times until we conclude—paradoxically—that the students know at the outset that the exam will not take place on the first day of term, either.

Williamson introduces a total of eight Glimpse‐like and four Surprise‐like paradoxes (p. 135 and ff). He argues that they all belong to the same family and that they should all be solved together. He writes:

[T]he pupils' reasoning is unsound in every case, and the cases are similar enough to make this unlikely to be mere coincidence. A common error should be sought. [A]ny diagnosis of one or more of the [Surprise‐like paradoxes] which does not extend to [the Glimpse‐like paradoxes], although perhaps correct as far as it goes, should be presumed incomplete, not having identified the common error […] any adequate diagnosis of the Surprise Examination should extend to the Glimpse. (Williamson, 2000, pp. 137–138)

In Williamson's view, an adequate diagnosis consistently blames the application of the KK principle, in both the Glimpse and the Surprise reasonings. However, Williamson's argument from analogy fails to convince.

First off, it should be noted that Lemma 1, Theorem 2, and Corollary 3 do not depend on a particular approach to the Surprise. These results establish that KK is both of no use and of no need when it comes to blocking the students' reasoning. But Williamson's insistence that the Surprise and the Glimpse are analogous obscures this fundamental fact: that basic results *already establish* what goes wrong in the surprise reasoning—namely, that the students mistakenly make use of KR in order to exclude Friday as a possible exam day.

In any event, there's several of reasons for thinking that, *pace* Williamson, any similarity between the Surprise and the Glimpse must be superficial. To begin with, the differences between the various Glimpse‐like reasonings Williamson considers, on the one hand, and the differences between various Surprise‐like paradoxes, on the other, are essentially cosmetic. All the Glimpse‐like paradoxes share the logical structure of the Glimpse; all the Surprise‐like paradoxes share the logical structure of the Surprise. The structures in question, however, are very different. If we narrow down our focus on the reasoning by means of which the students rule out a given day of the week, the structure of the Glimpse, pictured two paragraphs back, is relatively simple. It's essentially a Sorites‐like reasoning, with a base step, that the exam will not take place on the last day of term, and a tolerance‐like principle, that is, the margin for error principle, that if it's known that the exam will not take place on day *i* from the end of term, then it will not take place on day *i* + 1 either. By contrast, the corresponding reasoning in the Surprise is significantly more complex:


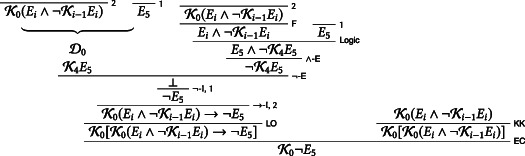


The reasonings are different because they make use of both different assumptions and different epistemic principles. For one thing, what the students know in the two scenarios is very different. In the Glimpse, the students know that the exam will not be near the end of the term; in the Surprise, they know that the exam will be a surprise, that is, they know that they will not know on the morning of the exam that the exam will take place on that day. For another, the base step in the Glimpse, viz. that the exam will not take place the last day of term, is directly established via the students' glimpse at the calendar. That is, the base step of the Glimpse is *known* and, just like the base step of the Sorites, it cannot be reasonably questioned. By contrast, since the publication of Quine's paper on the Surprise, the base step in the Surprise, viz. that the exam will not take place on Friday, *can* be reasonably questioned. Indeed, as we have argued, it should, being as it is the result of a complex, arguably fallacious, reasoning.^8^

Finally, Williamson writes that “the teacher's announcement [in the Surprise Examination paradox] corresponds to the claim in the Glimpse that if the examination is *i* + 1 days from the end, then the pupils do not know that it is not *i* days from the end” (pp. 138–139). However, the teacher's announcement is of the form $E_{i}\wedge \neg K_{i-1}E_{i}$, whereas the margin for error in the Glimpse is of the form $E_{i-1}\to \neg K\neg E_{i}$ or, equivalently, $K\neg E_{i}\to \neg E_{i-1}$. Obviously, these two claims have different logical forms. More generally, unlike the Glimpse, the Surprise reasoning involves *no* margin for error principle and requires no commitment to a particular conception of knowledge. Conversely, unlike the Surprise, the Glimpse involves no knowledge retention principle.

While we admit no knockdown argument, these considerations cast serious doubts on Williamson's contention that the Surprise and the Glimpse belong to a common genus. Worse still, we suspect that blaming KK principle does not yield a correct approach to the Glimpse in the first place. Consider a version of the Glimpse in which the students' glimpse to the calendar only reveals that the exam will take place towards the middle of the following *week*, that is, at some point between Tuesday and Thursday. Arguably, this constitutes inexact knowledge of the exam's date, on Williamson's understanding of the notion. However, it would be a mistake to accept that if the students know that the exam will not take place on Friday next week, the exam will not take place on Thursday either—since, we are assuming, the exam may well take place on Thursday. But, then, the relevant instance of the margin for error principle here is clearly false. And, we submit, if it's false in the one‐week version of the Glimpse, it's hard to see why we should accept it in the original version.^9^

## 5 CONCLUDING REMARKS

The Surprise and Williamson's Glimpse are loosely similar reasonings: both make use of the KK principle to fallaciously eliminate an arbitrary number of days as possible exam days. Yet, we have argued, this is where the similarities end. While both reasonings make use of KK, their premises and structure are different. Moreover, there is *no need* to invoke KK in order to provide an adequate diagnosis of the surprise examination paradox: it can be shown that the knowledge retention principle already commits the students to an outright inconsistency (if the exam is given on Friday). And, *pace* Williamson, it is also of *no use* to invalidate KK in the surprise reasoning: if no exam is given by Thursday, the students will have derived a falsehood—that there will be no exam on Friday—without making use of such a principle. Indeed, we have suggested, there's reasons for doubting the truth of the margin for error principles on which Williamson bases his diagnosis of the Glimpse. For all the Surprise and the Glimpse tell us, KK is innocent.

## ENDNOTES

^1^ We should clarify at the outset that our aim here is not so much to defend KK, as to set the record straight: if KK is false (and, for what is worth, we suspect it is), this is not because of the role it plays in paradoxical reasonings such as the Surprise and the Glimpse. For some recent defences of KK, see Greco (2014), Das and Salow (2018), and Stalnaker (2015).

^2^ Wright and Sudbury (1977) also offer a technically rigorous presentation of the paradox. Their diagnosis agrees with Kripke's, and ours, that the culprit is to be individuated with the assumption that the students' positive epistemic status towards the teacher's announcement is not lost throughout the week (this is principle *d(iv)* in Wright and Sudbury's terminology, see p. 53 of their paper), rather than on some KK‐like principle. The main difference with our presentation and Wright and Sudbury's is that, following Kripke, we frame the notion of surprise in terms of knowledge, whereas they frame it in terms of “reasonable belief” (so Wright and Sudbury should be credited with making the largely unappreciated point that the factivity of knowledge is not required by the students' reasoning). We should notice, though, that Wright and Sudbury's notion of “reasonable belief” seems stronger than the ordinary notion of “justified belief.” In particular, Wright and Sudbury (1977, p. 49) assume that one cannot *reasonably* believe both a sentence *P* and its negation (this assumption is required for them to show that the teacher's announcement is a blindspot of sorts, that cannot be reasonably believed after the second last day of week if no exam has been given yet by that time). However, it is sometimes argued that one can be *justified* in believing both *P* and ¬*P*. For instance, on certain views of the Preface Paradox, an author can be justified in believing that the claims made in one's latest book are all true (since each claim has been well‐researched) *and* that some such claim is false (since the author is modest and even the best books contain mistakes). Here we follow Kripke's lead and express surprise using the notion of knowledge, for essentially two reasons. The first is that this allows us to better evaluate Williamson's parallelism between the Surprise and the Glimpse, which also involves knowledge. The second is that the use of the knowledge operator allows us to explain why, as a matter of (epistemic) logic, the teacher's announcement must be lost on the second last day of the week, if no exam has been given by then, without having to rely on Wright and Sudbury's assumption that one can only reasonably believe a sentence and its negation on pain of inconsistency. As our presentation will make clear, this explains a number of features of the puzzle. More specifically, it explains why (i) the students' knowledge of the teacher's announcement cannot be retained throughout the week, (ii) the students can still be surprised if the week is only 1‐day long, and (iii) the teacher's announcement can be true irrespective of the length of the week (Wright and Sudbury make points that are analogous to (i) and (ii) but, to our knowledge, do not make point (iii)).

^3^ Thus, this is shorthand for $\left(E_{1}\wedge \neg K_{0}E_{1}\right)\vee \left(E_{2}\wedge \neg K_{1}E_{2}\right)\vee \ldots \vee \left(E_{5}\wedge \neg K_{4}E_{5}\right).$

^4^ We should also note that, as Williamson (2000, p. 140) observes, the full power of KK isn't actually needed to run the above version of the paradox: a capped KK holding up to five iterations of K, but not more, would suffice. To our minds, such a capped principle is plausible enough. At any rate, as we argue in §§3–4 below, neither KK nor its much weaker capped counterpart are required in order to reach a paradoxical conclusion via the Surprise Exam reasoning. For the sake of simplicity, we focus on KK throughout, even though everything we say already applies to its weaker, capped version.

^5^ Fitch credits the reasoning to an anonymous reviewer, who was later discovered by Joe Salerno (and by one of the present authors) to be Alonzo Church (for details, see Salerno, 2009).

^6^ As a referee has pointed out, the situation here is analogous to a 2‐day version of the paradox, in which the surprise exam is announced, say on Wednesday, to take place between Thursday and Friday. The 2‐day version is not different from the 5‐day version we are considering: in each case, the teacher's announcement can be true and the students can be surprised on any day of the week. In particular, in the 2‐day case, the students can be surprised on Friday, since, if no exam is given on Thursday, by then they will have lost knowledge of the teacher's announcement; and they can be surprised on Thursday, because, if the exam is given on Thursday, they will not know on Wednesday that the exam will take place on Thusday.

^7^ To be sure, one can modify the original setting in such a way that the students *have* reasons for privileging one part of the teacher's announcement at the expenses of the other—indeed, Wright and Sudbury (1977, pp. 54–55) precisely discuss such modified scenarios and their implications. However, the existence of such scenarios does not alter the fact that, absent reasons for privileging one part of the teacher's announcement, it is ceteris paribus a fallacy to favour the exam component over the surprise component.

^8^ We're indebted to an anonymous referee for helping us appreciate this further difference between the Surprise and the Glimpse.

^9^ For an argument to the effect that, contrary to what's assumed in the Glimpse, the margin for error principle is not known, see Stalnaker (2009, §3). For a more direct argument against the margin for error principle, see Stalnaker (2015, p. 33 and ff.).

## APPENDIX A.

We briefly show that there's models of all the principles used by the students that also invalidate the knowledge retention principle KR. That is, the problematic KR is not “hidden” within the principles accepted by the students and invalidating KR suffices to restore consistency.

### 1 MODELS

We work with standard Kripkean modal semantics and some elements of Priorean temporal semantics. A model for our language is a quituple $ℳ=〈W,T,≺,ℛ,I〉$ such that:

1. *W* is a non‐empty set of possible worlds w.
2. T is a set of times *t*_*i*_ such that *i* ∈ ℕ_0_.
3. ≺ ⊆T×T is an ordering of T with the following features:
  - Irreflexivity: For all times t∈T: 〈*t*, *t*〉∉ ≺
  - Transitivity: For all times $t,t^{\prime },t^{\prime \prime }\in T$: If 〈*t*, *t*′〉∈ ≺ and 〈*t*′, *t*′′〉∈ ≺, then 〈*t*, *t*′′〉∈ ≺.
  - Asymmetry: For all times $t,t^{\prime }\in T$: If 〈*t*, *t*′〉∈ ≺, then it is not the case that 〈*t*′, *t*〉∈ ≺.
  - Totality: For all times $t,t^{\prime }\in T$: Either 〈*t*, *t*′〉∈ ≺, or 〈*t*′, *t*〉∈ ≺, or *t* = *t*′.
4. The accessibility relation ℛ⊆W×T×W is reflexive, in the following sense:
  For all *w* ∈ *W* and all t∈T: 〈*w*, *t*, *w*〉 ∈ ℛ
5. *I* is the following interpretation function: $\left\{E\right\}\times W\times T\mapsto \left\{0,1\right\}$.

The important point to notice here is that, contrary to standard modal semantics, ℛ is sensitive to different times. This is required because we use ℛ to define the knowledge operator K to be introduced below (and knowledge can be gained at a certain time and lost at some later time).

### 2 TRUTH CONDITIONS

The valuation function *v* relative to a model ℳ and a *w* ∈ *W* is standard, but we must also account for the different times of evaluation. One way to do it is to use the following clause for atomic formulae:

*v*_ℳ_(E_*i*_, *w*) = 1 iff *I*(E, *w*, *t*_*i*_) = 1 for every *i* ∈ ℕ_0_ such that $t_{i}\in T$.

Since knowledge is also relative to time, we can use the following definition for the *K*‐operator:

$v_{ℳ}\left(K_{i}\phi ,w\right)=1$ iff for all *w*^′^ ∈ *W* such that 〈*w*, *t*_*i*_, *w*^′^〉 ∈ ℛ: *v*_ℳ_(*ϕ*, *w*^′^) = 1 for every *i* ∈ ℕ_0_ such that $t_{i}\in T$.

### 3 THE DESIRED MODEL

Here we give a model and a world of evaluation which validate all principles used in the students' reasoning, but invalidate KR. Let the world of evaluation be *w*_0_. Then the model ${ℳ}_{D}=〈W_{D},T_{D},{≺}_{D},{ℛ}_{D},I_{D}〉$ is constructed as follows:

- *W*_*D*_ = {*w*_0_, *w*_1_}
- $T_{D}=\left\{t_{0},t_{1},t_{2},t_{3},t_{4},t_{5}\right\}$
- ≺_*D*_ ={〈*t*_0_, *t*_1_〉, 〈*t*_0_, *t*_2_〉, 〈*t*_0_, *t*_3_〉, 〈*t*_0_, *t*_4_〉, 〈*t*_0_, *t*_5_〉, 〈*t*_1_, *t*_2_〉, 〈*t*_1_, *t*_3_〉, 〈*t*_1_, *t*_4_〉, 〈*t*_1_, *t*_5_〉, 〈*t*_2_, *t*_3_〉, 〈*t*_2_, *t*_4_〉, 〈*t*_2_, *t*_5_〉, 〈*t*_3_, *t*_4_〉, 〈*t*_3_, *t*_5_〉, 〈*t*_4_, *t*_5_〉}
- ℛ_*D*_ = {〈*w*_0_, *t*_0_, *w*_0_〉, 〈*w*_0_, *t*_1_, *w*_0_〉, 〈*w*_0_, *t*_2_, *w*_0_〉, 〈*w*_0_, *t*_3_, *w*_0_〉, 〈*w*_0_, *t*_4_, *w*_0_〉, 〈*w*_0_, *t*_5_, *w*_0_〉, 〈*w*_1_, *t*_0_, *w*_1_〉, 〈*w*_1_, *t*_1_, *w*_1_〉, 〈*w*_1_, *t*_2_, *w*_1_〉, 〈*w*_1_, *t*_3_, *w*_1_〉, 〈*w*_1_, *t*_4_, *w*_1_〉, 〈*w*_1_, *t*_5_, *w*_1_〉, 〈*w*_0_, *t*_4_, *w*_1_〉}
- *I*_*D*_ is such that
  - *I*_*D*_(*E*, *w*_0_, *t*_0_) = *I*_*D*_(*E*, *w*_0_, *t*_1_) = *I*_*D*_(*E*, *w*_0_, *t*_2_) = *I*_*D*_(*E*, *w*_0_, *t*_3_) = *I*_*D*_(*E*, *w*_0_, *t*_4_) = 0 and *I*_*D*_(*E*, *w*_0_, *t*_5_) = 1,
  - *I*_*D*_(*E*, *w*_1_, *t*_0_) = *I*_*D*_(*E*, *w*_1_, *t*_1_) = *I*_*D*_(*E*, *w*_1_, *t*_2_) = *I*_*D*_(*E*, *w*_1_, *t*_3_) = *I*_*D*_(*E*, *w*_1_, *t*_4_) = *I*_*D*_(*E*, *w*_1_, *t*_5_) = 0.

It can be shown that this model invalidates KR, but validates all the other principles used by the students, including the students' knowledge of the teacher's announcement $K_{0}\left(\left(E_{1}\wedge \neg K_{0}E_{1}\right)\vee \left(E_{2}\wedge \neg K_{1}E_{2}\right)\vee \left(E_{3}\wedge \neg K_{2}E_{3}\right)\vee \left(E_{4}\wedge \neg K_{3}E_{4}\right)\vee \left(E_{5}\wedge \neg K_{4}E_{5}\right)\right)$ and the empirical premise (¬*E*_1_ ∧  ¬ *E*_2_ ∧  ¬ *E*_3_ ∧  ¬ *E*_4_). For reasons of space, here we only prove that KR fails in ℳ_*D*_ at *w*_0_. To provide a counterexample to KR we must find a *ϕ*, a $t_{i}\in T_{D}$ and a $t_{j}\in T_{D}$ such that 〈*t*_*i*_, *t*_*j*_〉 ∈ ≺, $v_{{ℳ}_{D}}\left(K_{i}\phi ,w_{0}\right)=1$, and $v_{{ℳ}_{D}}\left(K_{j}\phi ,w_{0}\right)=0$. Now let *ϕ* be (*E*_1_ ∨ *E*_2_ ∨ *E*_3_ ∨ *E*_4_ ∨ *E*_5_). Moreover, let *t*_*i*_ be *t*_0_ and let *t*_*j*_ be *t*_4_. We can then reason as follows. Since *I*_*D*_(*E*, *w*_0_, *t*_5_) = 1 it follows that $v_{{ℳ}_{D}}\left(E_{5},w_{0}\right)=1$ wherefore $v_{{ℳ}_{D}}\left(\left(E_{1}\vee E_{2}\vee E_{3}\vee E_{4}\vee E_{5}\right),w_{0}\right)=1$. Because *w*_0_ itself is the only *w*^′^ ∈ *W*_*D*_ such that 〈*w*_0_, *t*_0_, *w*^′^〉 ∈ ℛ_*D*_ it follows that $v_{{ℳ}_{D}}\left(K_{0}\left(E_{1}\vee E_{2}\vee E_{3}\vee E_{4}\vee E_{5}\right),w_{0}\right)=1$. On the other hand, since *I*_*D*_(*E*, *w*_1_, *t*_0_) = *I*_*D*_(*E*, *w*_1_, *t*_1_) = *I*_*D*_(*E*, *w*_1_, *t*_2_) = *I*_*D*_(*E*, *w*_1_, *t*_3_) = *I*_*D*_(*E*, *w*_1_, *t*_4_) = *I*_*D*_(*E*, *w*_1_, *t*_5_) = 0, it follows that $v_{{ℳ}_{D}}\left(\left(E_{1}\vee E_{2}\vee E_{3}\vee E_{4}\vee E_{5}\right),w_{1}\right)=0$. Because 〈*w*_0_, *t*_4_, *w*_1_〉 ∈ ℛ_*D*_ we conclude that $v_{{ℳ}_{D}}\left(K_{4}\left(E_{1}\vee E_{2}\vee E_{3}\vee E_{4}\vee E_{5}\right),w_{0}\right)=0$. Thus, KR is invalid.
